# Association between bedtime snacking and subclinical hypothyroidism

**DOI:** 10.1265/ehpm.25-00419

**Published:** 2026-03-13

**Authors:** Yuji Shimizu, Nagisa Sasaki, Asuka Oyama, Yuko Noguchi, Mutsumi Matsuu-Matsuyama, Koichiro Hamada, Shin-Ya Kawashiri, Hirotomo Yamanashi, Seiko Nakamichi, Yasuhiro Nagata, Takahiro Maeda, Naomi Hayashida

**Affiliations:** 1 Department of General Medicine, Nagasaki University Graduate School of Biomedical Sciences, Nagasaki, Japan; 2 Epidemiology Section, Division of Public Health, Osaka Institute of Public Health, Osaka, Japan; 3 Department of Community Medicine, Nagasaki University Graduate School of Biomedical Sciences, Nagasaki, Japan; 4 Division of Strategic Collaborative Research, Atomic Bomb Disease Institute, Nagasaki University, Nagasaki, Japan; 5 Leading Medical Research Core Unit, Nagasaki University Graduate School of Biomedical Sciences, Nagasaki, Japan; 6 Nagasaki University Health Center, Nagasaki, Japan

**Keywords:** Bedtime snacking, Endothelial repair, Euthyroid population, Subclinical hypothyroidism

## Abstract

**Background:**

Thyroid hormones support endothelial repair, whereas bedtime snacking is linked to a higher risk of chronic kidney disease (CKD). Since endothelial dysfunction is a core feature of CKD, bedtime snacking could potentially contribute to subclinical hypothyroidism (SCH) by elevating the demand for endothelial repair. This study aimed to explore the association between bedtime snacking and SCH.

**Method:**

In this cross-sectional study, 1,478 Japanese individuals aged 40–69 years with normal thyroid function were enrolled; normal thyroid function was defined as free triiodothyronine (T3) and free thyroxine (T4) levels within the reference ranges and the absence of thyroid-related medication use. Individuals with elevated serum concentrations of TSH (>4.01 µIU/mL) were defined as having SCH. Bedtime snacking was determined in the basis of participants’ affirmation to the question “Do you consume a night meal or snack after dinner, within two hours of bedtime, three or more times per week? (Yes, No)”.

**Results:**

In the study population, 263 individuals reported a bedtime snacking habit, whereas SCH was identified in 81 individuals. A statistically significant association was found between bedtime snacking and SCH. The sex- and age-adjusted odds ratios (OR) and 95% confidence intervals (CI) were 1.77 (1.05, 2.99). This association remained significant after additional adjustment for skipping breakfast and late dinner; 1.83 (1.07, 3.11), and further adjustment for free T4, atherosclerosis, hypertension, diabetes, CKD, and thyroid cysts; 1.93 (1.11, 3.35), respectively.

**Conclusion:**

Bedtime snacking is positively associated with SCH, potentially due to an increased physiological demand for endothelial repair. This finding is not only an efficient tool for diagnosing the early stages of endothelial and thyroid dysfunction, but also for clarifying the mechanism underlying the regulation of thyroid hormones related to endothelial health.

## Graphical Abstract

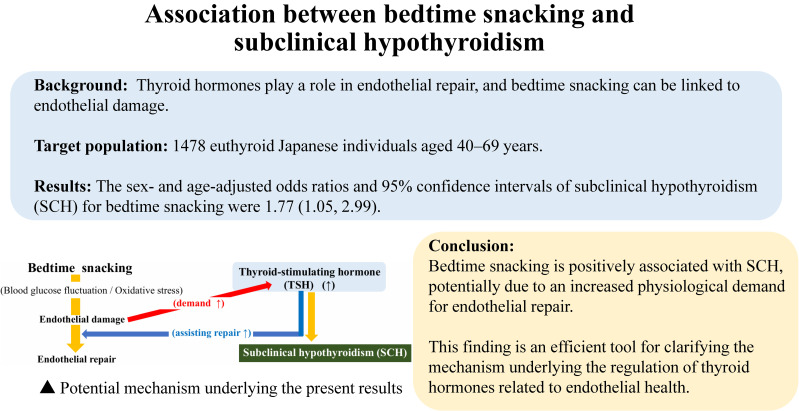

## Introduction

Oxidative stress is significantly associated with thyroid function [1], and both hypoxia and elevated oxidative stress are key contributors to endothelial dysfunction [2, 3].

Subclinical hypothyroidism (SCH), defined by elevated serum thyroid-stimulating hormone (TSH) levels in the presence of normal circulating thyroid hormone concentrations, has been reported to be associated with a decreased number of endothelial progenitor cells. Hormone replacement therapy increases endothelial progenitor cells [4], suggesting that thyroid hormones contribute to endothelial repair.

With advancing age, levels of hypoxia and oxidative stress increase in the body [5–7]. Consequently, the demand for endothelial repair processes in which thyroid hormones play a key role is likely higher in older adults than in young individuals. Differences in the requirement for endothelial repair may, at least in part, account for the higher prevalence of SCH observed in older adults compared with younger individuals [8]. These studies partly suggest that assessments of thyroid function should consider not only circulating hormone levels but also the physiological demand for thyroid hormones.

However, in routine clinical practice, thyroid hormone requirements are generally not considered when evaluating thyroid function. Identifying lifestyle-related factors that are significantly associated with SCH can provide valuable insights into the physiological demand for thyroid hormones in daily life.

Given that endothelial dysfunction is recognized as a contributing factor to chronic kidney disease (CKD) [9], and that bedtime snacking has been reported to increase CKD risk among Japanese individuals [10], habitual bedtime snacking may constitute a notable risk factor for SCH.

Evaluating the association between bedtime snacking and SCH not only clarifies the lifestyle-related risk of the condition but is also an efficient tool for understanding the mechanism of SCH.

The Clinical Guidelines Committee of the Japan Thyroid Association reports that the prevalence of SCH in the general Japanese population ranges from 3.3% to 10.2% [11]. Because ethnic differences are known to influence thyroid function [12], Japan’s largely monoethnic population provides an appropriate setting for investigating the association between bedtime snacking and SCH.

To investigate the association between bedtime snacking and SCH, a cross-sectional study was conducted among Japanese adults with normal thyroid hormone levels, defined as free triiodothyronine (T3) and free thyroxine (T4) within the reference range. In this study, individuals who consumed a meal or snack after dinner within two hours before bedtime on three or more days per week were classified as bedtime snack eaters.

## Methods

Methods pertaining to the present risk assessments, including evaluations of thyroid function, renal function, and carotid intima–media thickness (CIMT), have been detailed in previous reports [13, 14].

### Study population

The study population comprised 1,582 Japanese adults aged 40–69 years living in Saza Town, Nagasaki Prefecture in western Japan, who participated in annual health examinations in 2014. To minimize the potential influence of thyroid-related conditions, we excluded individuals undergoing treatment for thyroid disorders (n = 25), those with missing thyroid function data (TSH, free T3, or free T4) (n = 16), and those with abnormal free T3 (reference range: 2.1–4.1 pg/mL) or free T4 (reference range: 1.0–1.7 ng/dL) levels (n = 60). Participants with missing blood pressure data (n = 1), serum creatinine measurements (n = 1), or information on eating habits (n = 1) were also excluded. Ultimately, 1,478 participants were included in the analysis, with a mean age of 58.4 years (standard deviation, 8.3 years; range, 40–69 years).

This study was approved by the Ethics Committee of the Nagasaki University Graduate School of Biomedical Sciences (project registration number: 14051404-15; approval date: June 8, 2023). Informed consent was obtained from all participants, and written consent forms were used to ensure that participants fully understood the study objectives. All procedures involving human participants adhered to the ethical standards of the institutional research committee and the principles of the 1964 Declaration of Helsinki and its subsequent amendments.

### Data collection and laboratory measurements

Trained interviewers obtained information on participants’ clinical characteristics, and fasting blood samples were collected from all participants before midday. All participants were instructed to finish dinner by 21:00 on the day preceding their medical examination.

Participants who answered “Yes” to the question “Do you consume a night meal or snack after dinner, within two hours of bedtime, three or more times per week? (Yes, No)” were classified as having a bedtime snack.

Participants who answered “Yes” to the question “Do you have dinner less than two hours before going to bed, three or more times a week? (Yes, No)” were classified as having late dinners.

Participants who answered “Yes” to the question, “Do you skip breakfast more than three times a week?” were classified as having a habit of skipping breakfast.

Blood pressure was measured in the right arm after at least 5 minutes of seated rest using an automated sphygmomanometer (HEM-907; Omron, Kyoto, Japan), and values were recorded by a trained observer. Hypertension was defined as systolic blood pressure ≥140 mmHg, diastolic blood pressure ≥90 mmHg, or current use of antihypertensive medication [15, 16].

Serum TSH, free T3, and free T4 concentrations were determined using chemiluminescent immunoassays (LSI Medience Corporation, Tokyo, Japan). Reference ranges for free T3 (2.1–4.1 pg/mL), free T4 (1.0–1.7 ng/dL), and TSH (0.39–4.01 µIU/mL) have been reported previously [17]. Subclinical hypothyroidism was defined as a TSH concentration >4.01 µIU/mL with normal free T3 and free T4 levels [18, 19].

Serum creatinine and glycated hemoglobin (HbA1c) levels were measured using standard laboratory procedures at SRL Inc. (Tokyo, Japan) [20, 21]. Estimated glomerular filtration rate (eGFR) was calculated using the equation proposed by the Japanese Chronic Kidney Disease Initiative working group [22]. CKD was defined as eGFR <60 mL/min/1.73 m^2^ [9, 13, 23]. In accordance with the criteria of the Japan Diabetes Society [24], diabetes was defined as HbA1c ≥6.5% or current use of glucose-lowering medication [25].

CIMT was assessed by ultrasonography of the left and right common carotid arteries, performed by an experienced vascular technician using a LOGIQ Book XP equipped with a 10-MHz transducer (GE Healthcare, Milwaukee, WI, USA). Maximum CIMT values for both sides were quantified using digital edge-detection software (Intimascope; MediaCross, Tokyo, Japan) in accordance with a previously described protocol [26]. Intimascope was developed to reduce measurement error and enables semi-automated identification of the internal and external vessel wall boundaries, providing distance measurements at sub-pixel resolution (approximately 0.01 mm) through a polynomial measurement algorithm [27]. Subclinical atherosclerosis was defined as CIMT ≥1.1 mm, consistent with prior studies [14, 28], which have reported CIMT values <1.1 mm to fall within the normal range [29].

In this study, thyroid cyst was defined as a structure with a maximum diameter of ≥2.0 mm containing no solid components, as observed in previous studies [30, 31].

### Statistical analysis

Sex-specific distributions of serum TSH concentrations were calculated.

Differences according to bedtime snacking status were also assessed. Data on sex, late dinner, skipping breakfast, atherosclerosis, hypertension, diabetes, CKD, and thyroid cysts are presented as percentages. Continuous variables such as age, free T3, free T4, and free T3 to free T4 ratio (FT3/FT4 ratio) are expressed as means (standard deviations).

Because TSH exhibited a skewed distribution, population characteristics are presented as medians with interquartile ranges, and TSH values were logarithmically transformed for analysis. Model adequacy was assessed using the Hosmer–Lemeshow goodness-of-fit test. Logistic regression analysis was performed to estimate odds ratios (ORs) and 95% confidence intervals (CIs) for SCH in relation to bedtime snacking.

Confounding factors were adjusted using four models. Model 1 was adjusted for sex and age, while Model 2 was further adjusted for eating-related behavior, which could influence bedtime snacking status, such as late dinner and skipping breakfast. As bedtime snacking was hypothesized to increase the demand for thyroid hormones, potentially leading to SCH without reducing thyroid function, in Model 3, free T4 was included as a confounder. Given that endothelial status potentially contributed to the association between skipping breakfast and SCH, endothelial-related factors associated with thyroid function were considered confounders in Model 4. Thyroid function activity evaluated by the deiodination marker, FT3/FT4 ratio, has been reported to be associated with CKD and SCH [13]. Thyroid cysts have been reported to modulate the association between TSH and renal function [30], and between TSH and hypertension [15]. Atherosclerosis has been identified as a determinant of the association between thyroid cysts and hypertension [16]. Furthermore, HbA1c levels are inversely associated with thyroid cysts [32]. SCH may influence the association between HbA1c and renal function [18]. The absence of thyroid cysts has been suggested to indicate latent thyroid injury [33]. These factors are likely intricately interconnected, collectively contributing to the mechanism underlying the association between bedtime snacking and SCH. Accordingly, in the further adjusted model (Model 4), in addition to the factors adjusted for in Model 3, atherosclerosis, hypertension, diabetes, CKD, and thyroid cysts were included as confounders.

All statistical analyses were conducted using SAS software for Windows (version 9.4; SAS Institute Inc., Cary, NC, USA). A two-sided p value <0.05 was considered to indicate statistical significance.

## Results

Among the study population, 263 individuals reported habitual bedtime snacking, and 81 were identified as having SCH.

### Characteristics of the study population

Table 1 displays the clinical characteristics of the study population according to bedtime snacking status. Compared with individuals who did not engage in bedtime snacking, those who did exhibited a significantly lower proportion of men and a lower prevalence of hypertension, were younger in age, and had a higher prevalence of late dinner consumption.

**Table 1:** Characteristics of the study population

	**Bedtime snacking**		***p-value***

	**(−)**	**(+)**	
Number of participants	1215	263	
Men, %	35.9	27.8	0.012
Age, years	58.9 (8.1)	56.0 (8.6)	<0.001
TSH, µIU/mL	1.5 [1.1, 2.2]*^1^	1.6 [1.1, 2.3]*^1^	0.784*^2^
free T3, pg/mL	3.2 (0.3)	3.2 (0.3)	0.497
free T4, nd/dL	1.3 (0.2)	1.2 (0.2)	0.199
FT3/FT4 ratio	2.57 (0.35)	2.59 (0.35)	0.543
Late dinner, %	11.4	25.1	<0.001
Skipping breakfast, %	9.5	13.3	0.061
Atherosclerosis, %	8.6	8.0	0.762
Hypertension, %	37.4	29.3	0.012
Diabetes, %	8.2	6.8	0.452
CKD, %	15.7	11.4	0.075
Thyroid cyst, %	31.4	33.5	0.524

TSH, thyroid-stimulating hormone; T3, triiodothyronine; T4, thyroxine; FT3/FT4 ratio, free T3 to free T4 ratio; CKD, chronic kidney disease;

Age, free T3, free T4, and FT3/FT4 ratio values are presented as mean (standard deviation). *1: Values are reported as median [the first quartile, third quartile]. *2 Logarithmic transformation was applied for the calculation of *p*-values.

**Fig. 1:**
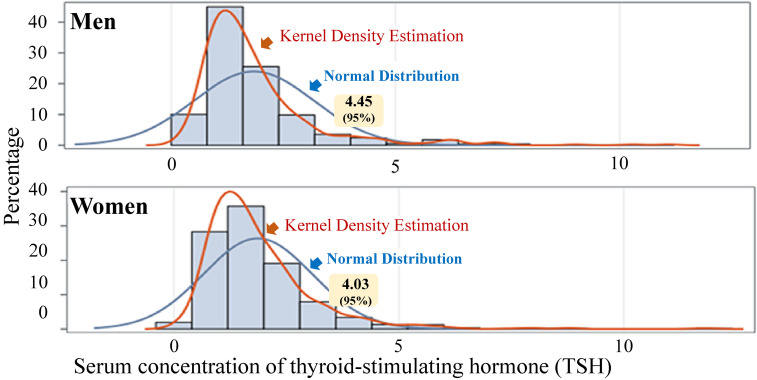
Distribution of serum concentration of TSH

Figure 1 shows the sex-specific serum TSH concentrations in the study population. Sex-specific median [the first quartile, third quartile] values (µIU/mL) of TSH were 1.49 [1.06, 2.15] for men and 1.58 [1.09, 2.29] for women. The 95% percent values of TSH were 4.45 µIU/mL for men and 4.03 µIU/mL for women.

### The association between bedtime snacking and SCH

In all the adjusted models, bedtime snacking was significantly and positively associated with SCH (Table 2). The fully adjusted OR (95% CIs) for bedtime snacking was 1.93 (1.11, 3.35).

**Table 2:** Association between bedtime snacking and SCH

	**Bedtime snacking**		***p*-value**

	**(−)**	**(+)**	
Number of participants	1215	263	
Number of SCH (%)	60 (4.9)	21 (8.0)	
Model 1	Reference	1.77 (1.05, 2.99)	0.036
Model 2	Reference	1.83 (1.07, 3.11)	0.026
Model 3	Reference	1.82 (1.06, 3.10)	0.029
Model 4	Reference	1.93 (1.11, 3.35)	0.019

SCH, subclinical hypothyroidism. Logistic regression model analysis was used to calculate the odds ratios and 95% confidence intervals for SCH in relation to bedtime snacking.

Model 1: adjusted for sex and age. Model 2: adjusted for sex, age, late dinner, and skipping breakfast. Model 3: adjusted for factors adjusted in Model 2 plus free thyroxine (T4). Model 4: adjusted for factors adjusted in Model 3 plus atherosclerosis, hypertension, diabetes, chronic kidney disease, and thyroid cysts.

### Sex-specific association between bedtime snacking and SCH

As presented in Table 3, the association between bedtime snacking and SCH was positive in both men and women. The fully adjusted ORs (95%CIs) for SCH associated with bedtime snacking were 2.10 (0.77, 5.72) in men and 1.90 (0.96, 3.73) in women.

**Table 3:** Sex-specific association between bedtime snacking and SCH

	**Bedtime snacking**		***p*-value**

	**(−)**	**(+)**	
Men			
Number of participants	436	73	
Number of SCH (%)	25 (5.7)	6 (8.2)	
Model 1	Reference	1.47 (0.58, 3.73)	0.418
Model 2	Reference	1.49 (0.58, 3.82)	0.407
Model 3	Reference	1.55 (0.60, 4.04)	0.366
Model 4	Reference	2.10 (0.77, 5.72)	0.147
Women			
Number of participants	779	190	
Number of SCH (%)	35 (4.5)	15 (7.9)	
Model 1	Reference	1.97 (1.04, 3.72)	0.038
Model 2	Reference	2.01 (1.04, 3.86)	0.037
Model 3	Reference	1.95 (1.01, 3.77)	0.047
Model 4	Reference	1.90 (0.96, 3.73)	0.064

SCH, subclinical hypothyroidism. Logistic regression model analysis was used to calculate the odds ratios and 95% confidence intervals for SCH in relation to bedtime snacking.

Model 1: adjusted for age. Model 2: adjusted for age, skipping breakfast, and late dinner. Model 3: adjusted for factors adjusted in Model 2 plus free thyroxine (T4). Model 4: Adjusted for factors adjusted in Model 3 plus atherosclerosis, hypertension, diabetes, chronic kidney disease, and thyroid cysts.

### Sensitivity analysis

Sensitivity analysis was performed using the 95th percentile of serum TSH concentrations as sex-specific cutoff values (4.45 µIU/mL for men and 4.03 µIU/mL for women) to classify SCH. The associations between bedtime snacking and SCH remained consistent. The odds ratios (95% confidence intervals) for SCH were 1.83 (1.07–3.12) in Model 1, 1.89 (1.09–3.25) in Model 2, 1.87 (1.08–3.25) in Model 3, and 2.00 (1.13–3.53) in Model 4.

## Discussion

The principal finding of this study was that bedtime snacking was positively associated with SCH.

To our knowledge, this is the first investigation to demonstrate a significant association between habitual bedtime snacking and SCH. As sex-specific analysis demonstrated essentially similar associations in men and women, sex differences did not appear to influence the relationship between bedtime snacking and SCH.

Previously, we reported a significant positive association between the FT3/FT4 ratio and SCH, as well as an inverse association between the FT3/FT4 ratio and CKD among Japanese individuals with normal free T3 and free T4 [13]. This previous study also revealed a positive association between SCH and CKD.

As the FT3/FT4 ratio is a recognized marker of deiodination, indicating the activity of thyroid hormones, individuals with elevated FT3/FT4 ratios are considered to exhibit greater thyroid hormone activity. A previous clinical study involving 100 patients with hypothyroidism and 20 healthy controls demonstrated that hypothyroidism is associated with reduced levels of endothelial progenitor cells, and that treatment with levothyroxine can improve the endothelial cell count [34]. Another controlled clinical trial involving individuals with SCH and healthy controls similarly reported that SCH was associated with a reduced endothelial progenitor cell count, and the T4 treatment increased this cell count [4].

The count of endothelial progenitor cells (CD34-positive cells) has been used as a marker of endothelial repair activity [35], reflecting their role in endothelial maintenance [36]. Taken together, contrasting patterns were observed for the FT3/FT4 ratio, with a positive association with SCH and an inverse association with CKD. These findings suggest that endothelial repair activity may be upregulated in individuals with SCH [13], while higher endothelial repair activity could exert a protective effect against CKD progression, given the established link between CKD and endothelial dysfunction [9]. However, despite the higher endothelial repair activity observed in individuals with SCH, the positive association between SCH and CKD indicates that these individuals experience more severe endothelial dysfunction than those without SCH. Therefore, increased demand for endothelial repair might cause SCH, and not only the activity of endothelial repair, but also the physiological demand of endothelial repair influences the FT3/FT4 ratio.

In contrast, the FT3/FT4 ratio did not differ significantly between individuals with and without bedtime snacking in the present study. In addition to endothelial repair activity, the demand for endothelial repair also influences the FT3/FT4 ratio. Therefore, the FT3/FT4 ratio was not significantly associated with bedtime snacking status.

A follow-up study of 316 Japanese middle-aged and older men reported that bedtime snacking was linked to an elevated risk of incident CKD [10]. This finding suggests that bedtime snacking may contribute to endothelial dysfunction.

These studies indicate that bedtime snacking may be detrimental to the endothelium and may induce SCH.

One retrospective study involving 1,062 normotensive older individuals reported that eating supper before bedtime served as a significant risk factor for the incidence of hypertension [37]. As hypertension is closely associated with endothelial dysfunction [38], eating a snack late at night might induce endothelial injury. However, in the present study, an association between bedtime snacking and SCH was observed even after adjusting for late dinners, hypertension, and atherosclerosis. Therefore, to elucidate the mechanism underlying the association between bedtime snacking and SCH, further investigation is necessary to clarify the effects of late dinner and bedtime snacking on endothelial function.

The clinical implication of this study is that bedtime snacking may be detrimental to the endothelium and may stimulate endothelial repair processes related to SCH. Thyroid function cannot be fully evaluated by serum thyroid hormone concentrations alone; the demand for thyroid hormones should also be considered. Therefore, this study is not only an efficient tool for diagnosing the early stages of endothelial and thyroid dysfunction, but also for clarifying the mechanisms underlying the regulation of thyroid hormones related to endothelial health.

Certain limitations of the present study should be recognized. Given the cross-sectional nature of this study, causality cannot be inferred. Bedtime snacking has been revealed to be associated with SCH in this study. However, the types of snacks associated with SCH have not yet been identified. Further studies investigating the content of snacks are necessary. In addition, the small sample size, particularly the limited number of men with SCH who reported bedtime snacking (n = 6), restricts the interpretability of the findings from analyses conducted among men. Studies with larger sample sizes are required to better elucidate the role of sex in the development of SCH.

## Conclusion

Taken together, these findings suggest that bedtime snacking is positively associated with SCH, possibly by promoting endothelial repair. Further investigation is necessary to evaluate the function of thyroid hormone; not only the serum concentration of thyroid hormone, but also the demand of thyroid hormone that relates to the status of the endothelium should be taken into consideration.

## Abbreviations

CKD: chronic kidney disease
SCH: subclinical hypothyroidism
T3: triiodothyronine
T4: thyroxine
OR: odds ratio
CI: confidence interval
TSH: thyroid-stimulating hormone
CIMT: carotid intima-media thickness
HbA1c: glycated hemoglobin
GFR: glomerular filtration rate
FT3/FT4 ratio: free T3 to free T4 ratio

## Declarations

### Availability of data and material

For the studies described in this review, we cannot publicly provide individual data because of participant confidentiality considerations according to the ethical guidelines in Japan. Additionally, obtaining informed consent does not include a provision for publicly sharing the data. Qualified researchers have access to minimal datasets. Please contact the Office of Data Management at ritouken@vc.fctv-net.jp. Information on the data request is available at http://www.med.nagasaki-u.ac.jp/cm/.
